# Editorial: The role of the bacteriome, mycobiome, archaeome and virome in animal health and disease

**DOI:** 10.3389/fvets.2022.1130187

**Published:** 2023-01-10

**Authors:** Mohamed Zeineldin, Ahmed Elolimy, Abdulrahman Alharthi, Mohamed Abdelmegeid

**Affiliations:** ^1^Department of Animal Medicine, College of Veterinary Medicine, Benha University, Banha, Egypt; ^2^Department of Animal Production, National Research Centre, Giza, Egypt; ^3^Department of Animal Production, College of Food and Agriculture Sciences, King Saud University, Riyadh, Saudi Arabia; ^4^Department of Animal Medicine, College of Veterinary Medicine, Kafr-Elsheikh University, Kafr El-Shaikh, Egypt; ^5^Higher Colleges of Technology, Abu Dhabi, United Arab Emirates

**Keywords:** microbiome, bacteriome, mycobiome, archaeome, virome, animal, health

The widespread of infectious diseases has shifted the focus of scientists to study the host–pathogen interaction to develop novel preventive and management strategies to overcome these diseases. Recently, the advancement in next generation sequencing technologies has shed the light on microbiome composition and function across a range of hosts and environments. Animal microbiome, including bacteria (bacteriome), fungi (mycobiome), archaea (archaeome), and viruses (virome) has become a growing area of research and increasingly recognized as an important driver of animal health and productivity. Therefore, understanding the complexity of animal microbiomes and its interaction with the host is key to develop strategies to improve animal health and production while reducing disease incidence.

Animal bacteriomes, collectively termed the microbiota, harbors complex and diverse microbial communities that reside in the lumen and mucosal surfaces at different body sites (1). Several studies have suggested that these complex microbial communities cooperate extensively with each other, and with their host, modulating host immune system, provide protection against pathogens as well as increase host tolerance to various stressors (2). Therefore, it is important to maintain the homeostasis of animal bacteriomes for proper health conditions as well as improving the clinical outcomes of infections. While the composition, function, and developmental dynamic of bacteriomes have been the focus of research in animals, other members of the animal microbiome are much less well-recognized and still in its infancy.

One of the major ubiquitous players of the microbiomes include the virome. The recent advancement in omics technology has facilitated the in-depth analysis of the entire animal virome, which, in turn, improves our understanding about the composition and function of viromes and its impact on animal health and disease (3). The animal viromes are composed of diverse and complex commensal and pathogenic viruses that infect cells of animal host, viruses that infect other members of animal microbiome (bacterial, fungal, and archaeal virome) and endogenous viral elements (3). The animal viromes inhabit all mucosal surfaces and persist systematically after viral infection clearance. Similar to bacteriome, the virome shapes the host immune system, playing a key role in the regulation of mucosal surface homeostasis and inflammation (4). In particular, the virome generates infectious viruses that continually shape the immunological phenotype of the host and stimulate the host immune response without causing clinical infections (5). Based on our information about the virome–host interactions, it is likely that variety of the virome lifecycle, allows the virome members to diversify and form mutualistic and sometimes symbiotic relationships with their host. Some evidence suggests that the relationship among viromes with other members of animal microbiomes “trans-kingdom relationships” as well as the systemic nature of the virome allows it to influence the host physiology and contributes to the state of health (4). However, this trans-kingdom relationship is still a largely unexplored area of animal viromes and warrants more exploration to determine the role of viromes in modulation of the compositions of other members of animal microbiomes.

Along with bacteriomes and viromes, the animals mycobiome and archaeome have been recently recognized as critical components in animal health despite being numerically inferior to the bacteriome (6, 7). It has been shown that animals mycobiome and archaeome are composed of various lineages, widely distributed and reveal body-site-specific patterns. Currently, mycobiome and archaeome in animals have been poorly investigated due to challenges in primer design, bioinformatics, well-curated database and lower DNA compared to bacteriomes. Despite these challenges, animal mycobiome and archaeome have been found to have complex interactions with other members of animal microbiome during ecosystem establishment and have implications on host health. Studies have also shown the ability of the mycobiome and archaeome to be manipulated by environmental factors, such as diet, more readily than the bacteriome, making it an excellent candidate for dietary interventions to promote animal growth and health (8). Further investigations into the mechanisms by which animal mycobiome and archaeome interact with other members of microbiome are warranted.

This Research Topic is aimed at collecting ongoing studies on the role of bacteriome, mycobiome, archaeome, and virome in animal health with an emphasis on their compositional changes associated with animal diseases (associative, correlative, or causal), and its potential role toward enhancing animal health and profitability.

In this Research Topic there are five papers covering the above mentioned aims.

The advancement in omics technology and bioinformatics allows the scientists to translate microbiome profiling into clinical applications and demonstrate disease causality (9). However, to distinguish causality and understand the host-microbiome relationship in disease pathophysiology, it is necessary to explore animal microbiome changes associated with disease processes. Understanding of the role of animal microbiome dysbiosis in disease pathogenesis may pave the way to the discovery of novel management strategies that can improve disease outcomes. In this Research Topic, Wu et al., described the alteration in the composition and diversity of gut microbiota associated with yak diarrhea. This study revealed that the alterations in gastrointestinal microbial composition and structure in diarrheal adult yaks may contribute to pathogen establishment and propagation in gastrointestinal tract. Similarly, Alsaaod, Weber et al., described the changes in microbiota compositions during bovine digital dermatitis and concluded that the *treponemes* and *Porphyromonas levii* constituting potential etiological agents in the development of “non-healing” claw horn lesions in cattle.

Animal microbiomes and their association with hosts can be affected by various abiotic and biotic factors, such as management strategies, diet composition, physiological conditions, stress, and disease states (1). Therefore, manipulation of animal microbiomes at different stages of the production cycle represents an attractive proposition for the animal production sector and has been suggested as a possible alternative to the use of antimicrobials in the livestock industry. Data from Varriale et al. showed that outdoor access periods with access to natural dietary sources increase the richness and diversity of cecal microbiota in chickens as well as increase the biosynthesis of micronutrients involved in vital cell processes. This study provided new insights into the impact of diet and environmental factors on the developmental dynamics of gastrointestinal microbial communities and predictions of metabolic functions in broiler chickens from a free-range system.

A new research horizon in medicine and agriculture revealed that modulation of specific members of the animal microbiome can optimize vital metabolic pathways and improve host phenotypes and productivity (10). Toward this end, a novel and underutilized approach employs administration of fecal microbiota transplant to modulate microbiome composition and improve host-mediated microbiome selection has been widely used in veterinary medicine (11). The aim of fecal microbiota transplant is to improve host performance *via* artificial transplant of the microbiome from healthy individuals. Ramírez et al., showed that giving fecal microbiota transplant to day-of-hatch chicks from healthy adults result in significantly higher body mass of birds and decreased residual feed intake, implying enhanced feed efficiency at 6 weeks of age. The results suggest that early-life microbiome transplants modulate market-relevant phenotypes in poultry and, thereby, may represent a significant advance toward microbiome-focused sustainable agriculture.

In summary, the results of the above mentioned studies revealed the role of animal microbiomes in animal health and disease. Despite all the existing studies related to animal microbiomes, the studies published in this Research Topic clearly show that there are still many aspects including the interplay between the microbiomes and the immune system to be studied.

## Author contributions

MZ, AE, AA, and MA wrote and edited the manuscript. All authors approved the submitted version.
